# The relation between athletic sports and prevalence of amenorrhea and oligomenorrhea in Iranian female athletes

**DOI:** 10.1186/1758-2555-1-16

**Published:** 2009-07-30

**Authors:** Haleh Dadgostar, Mohammad Razi, Ashraf Aleyasin, Talia Alenabi, Saeideh Dahaghin

**Affiliations:** 1 Sport Medicine Department of Iran Medical University. Rassole Akram Hospital., Iran University, Tehran, Iran; 2 Orthopaedic and Sport Medicine Department of Iran Medical University. Rassole Akram Hospital., Iran University, Tehran, Iran; 3 Gynaecologic and Obstetrics Department of Tehran Medical University. Shariati Hospital. Tehran University, Tehran, Iran; 4 Sport Medicine Federation, Islamic Republic of Iran. Tehran. Iran; 5 Rheumatology Research Center, Shariati Hospital, Tehran University, Tehran, Iran

## Abstract

**Background:**

In 1992, the concept of female athlete triad was introduced to describe the interrelated problems of amenorrhea, eating disorders and osteoporosis seen in female athletes. To gain a clearer picture of amenorrhea/oligomenorrhea in Iran, one of the main components of the female athlete triad, we therefore established this study on the prevalence of amenorrhea/oligomenorrhea in elite Iranian female athletes, also evaluating the risk factors of these disorders in the same population.

**Methods:**

This study performed as a cross-sectional study. All elite Iranian female athletes of 34 sports federation, including female athletes in national teams and medalists of Tehran were invited to participate. A total of 788 (95% response rate) returned the questionnaires and were examined. Younger athletes under the age of menarche were excluded. Each athlete completed a self-administered questionnaire, which covered the following questions about participant's demographic information, athletic history, history of injuries and menstrual pattern. In order to diagnose the causes of amenorrhea/Oligomenorrhea including polycystic ovary syndrome(PCOS), participants with amenorrhea/Oligomenorrhea underwent further investigation. They were evaluated by following Para clinic investigation, and an ultrasonographic study of ovary.

**Results:**

The age ranged from 13–37 (mean = 21.1, SD = 4.5). Seventy one (9.0%) individuals had amenorrhea/oligomenorrhea, among those, 11 (15.5%) had PCOS.

There was also a positive association between amenorrhea/oligomenorrhea and the following: age under 20 OR; 2.67, 95%CI(1.47 – 4.85), weight class sports OR; 2.09, 95%CI(1.15 – 3.82), endurance sports OR; 2.89, 95%CI(1.22 – 6.84), late onset of menarche OR; 3.32 95%CI(1.04–10.51), and use of oral contraceptive pills OR; 6.17, 95%CI(3.00 – 12.69). Intensity of training sport or BMI were not risk factors.

**Conclusion:**

These findings support the previous findings in the literature that the prevalence of amenorrhea/oligomenorrhea is high in athletes. Furthermore, we provided the first report on the prevalence of PCOS in female athletes with amenorrhea/oligomenorrhea. Athletes would be greatly benefited by greater general awareness about the complications of amenorrhea/oligomenorrhea.

To increase awareness of exercise-associated menstrual cycle irregularities, it is necessary to design complete and comprehensive education programs for female athletes, their parents, their coaches, and the relevant authorities.

## Background

Menstrual disorders such as amenorrhea/oligomenorrhea depend on many factors, including race, genetic make-up, BMI, and family history. However, the literature also shows that amenorrhea/oligomenorrhea is more prevalent among athletes than in the general population [1,2]. Despite discrepancies in the reported frequencies, which are attributable mainly to different definitions of amenorrhea/oligomenorrhea, unreported minor menstrual irregularities, and selection bias [2], this is commonly accepted.

To our knowledge, no study has evaluated amenorrhea/oligomenorrhea in Iranian female athletes, a group that is particularly interesting because the nature and practice of women's sports differ from those in the majority of other countries. This is due partly to the fact that women's professional sports – in the sense that sport is a woman's main profession – have almost no place in Iran.

More importantly, however, it is a consequence of the religious dress code after Iran's 1979 Islamic revolution, which requires women to cover all their hair, and to wear long, loose-fitting clothes that disguise the shape of their bodies. The code has presented Iranian sportswomen with various difficulties: those competing in international events have to respect it just as much as they respect rules of their sport. Sports that require tight-fitting clothing – for example, ballet, figure skating, swimming and synchronized swimming – are not open to them at all.

The relevance of their position becomes clear when juxtaposed with the Stand Position classification of the American College of Sports Medicine [3], according to which the risk of menstrual disorders is higher in Sports in which performance is subjectively scored or athletes who wear body-contour-revealing clothing, are categorized on the basis of their body weight, and practice endurance sports the sports that are represented less in Iran.

In view of these differences, we wished to establish whether the restrictions that apply to female athletes in Iran influence the frequency and risk factors of amenorrhea/oligomenorrhea relative to those in other countries. Even though female sport is much less professionalized in Iran than elsewhere, competitive sports are becoming increasingly important, as evidenced by women's presence in national competitions and in Islamic and few international games.

Since exercise-associated menstrual cycle irregularities are a threat to athletes' health, appropriate policy-making is required on the part of the sport authorities, especially those involved in women's sports. In 1992, the concept of female athlete triad was introduced to describe the interrelated problems of amenorrhea, eating disorders and osteoporosis seen in female athletes [4]. To gain a clearer picture of amenorrhea/oligomenorrhea, one of the main components of the female athlete triad, we therefore established this study on the prevalence of amenorrhea/oligomenorrhea in elite Iranian female athletes, also evaluating the risk factors of these disorders in the same population.

## Methods

### Study population

This study performed as a cross-sectional study, which took place in 2007–2008. All elite Iranian female athletes of 34 sports federation, including female athletes in national teams and Tehran's medalists were invited to participate in this study. Written informed consent was obtained from all participants.

To include all possible female athletes, we acquired the time-tables of the national sports' camps in different fields of sport and arranged for a visit. We also arranged an appointment with Tehran's medalists after contacting their coaches via written letters. A pre-designed structured questionnaire was handed to participants before/after training in the sport halls. A total of 830 participants were invited of whom 788 (95%) returned the questionnaires and were examined. Younger athletes under the age of menarche were excluded from this study.

### Measurements

Three specialists in sports medicine evaluated a pre-designed questionnaire. After conduction of a pilot study and implementation of necessary changes in the questionnaire, it has been used for the present study. This questionnaire covers the following questions about participant's demographic information, athletic history, history of injuries and menstrual pattern. Menstrual pattern includes information about the age of menarche, between cycles' gap, number of cycles in a year, and the longest gap between two cycles in the previous year. Details on history of menstrual dysfunction and any use of hormonal medicine in last year were also recorded. After filling questionnaires by participants, the questionnaire was scanned and controlled for any error or missing item by responsible physician (HD). She (HD) also examined all participants for presence of acne or hirsutism. The questions regarding menstrual pattern were specifically checked in the case of any uncertainty, and re-enquired.

In order to diagnose the causes of amenorrhea/Oligomenorrhea including hyperprolactinemia, hypothyroidism, and polycystic ovary disorders [PCOS]), participants with amenorrhea/Oligomenorrhea underwent further investigation. They were evaluated by following Para clinic investigation; measurements of Serum follicle stimulating hormone (FSH), luteinizing hormone (LH), Thyroid Stimulating Hormone (TSH), Prolactin, Free T4, Serum testosterone and Dehydroepiandrosterone (DHEA) and an ultrasonographic study of ovary.

#### Demographic characteristics

Age, education, height and weight were recorded on the questionnaire. Participants were assessed in two age categories; under the age of 20 and over 20 years old. BMI was calculated as weight in kilograms/height in square meters and was evaluated in three categories; BMI less than 20, BMI = 20 to 25 (normal group as the reference group) and BMI over 25. Education were evaluated in three categories; High school (under graduate), High school graduated and Bachelor degree.

#### Primary amenorrhea

menarche has not been occurred by age 14 in the absence of secondary sexual characteristics, or by 16 years of age in the presence of normal growth and secondary sexual characteristics[5].

#### Secondary amenorrhea

Menses have ceased at a point in time beyond establishment of regular menstrual cycles. In this study, secondary amenorrhea described as absence of at least 3 to 6 consecutive menstrual cycles or four or fewer menstrual period per year.[5]

#### Oligomenorrhea

Menstrual cycles fewer than 8 cycles per year, or the duration of the cycle exceeds 35 days [5].

#### Late-onset menarche

Menarche occurred after the age of 16 [5].

#### Polycystic ovary syndrome (PCOS)

The 2003 Rotterdam consensus workshop concluded that PCOS is a syndrome of ovarian dysfunction along with the cardinal features; hyperandrogenism and poly cystic ovary morphology. PCOS is diagnosed with presence of two or three following criteria (a) chronic anovulation or oligo-ovulation,(b) biochemical or clinical evidence of androgen excess, and (c) presence of polycystic ovaries on ultrasound [6].

#### Acne

is a chronic inflammatory disease of the Pilosebaceous Unit. It is characterized by Seborrhoea, the formation of comedones, erythematous papules and pustules, less frequency by nodules, deep postules or pseudocysts and in some cases is accompanied by scarring [7].

#### Hirsutism

Hirsutism in women defined as excessive thick (terminal) hair growth in facial and body regions [8].

### Drug intake

Participants were asked to record regular use of any medicine in the past year. Medicines were evaluated in three groups: 1-Oral contraceptives pills (OCPs), 2-Diuretics and/or weight-reducing drugs, 3-Thyroid hormones.

### Sports classification

According to the Stand position classification of American College of Sports Medicine potentially all physically active girls and women could be at risk for developing one or more components of the Triad (amenorrhea/oligomenorrhea, eating disorders and osteoporosis). Participation in sports that emphasize low body weight can be a risk factor. Those sports include:

1. Sports in which performance is subjectively scored (dance, figure skating, diving, gymnastics, aerobics). 2. Endurance sports emphasizing a low body weight (distance running, cycling, cross-country skiing). 3. Sports requiring body contour-revealing clothing for competition (volleyball, swimming, diving, cross-country running, cross-country skiing, and track) 4. Sports using weight categories for participation (horse racing, some martial arts, wrestling, rowing). 5. Sports emphasizing a prepubertal body habitus for performance success (figure skating, gymnastics, diving) [3].

Sport intensity: Intensity of sport were calculated as hours of training sport in a week (Hours per week).

### Statistical analysis

Quantitative data were described by mean and standard deviation and qualitative data by frequency. T-test and chi-square were used to compare quantitative and qualitative data respectively. Fischer's test was used when appropriate. Univariate logistic regression analysis was used initially to examine associations between Amenorrhea/Oligomenorrhea and potential risk factor. The following independent variables were evaluated in the univariate model separately: age, age of menarche, BMI, use of steroidal hormones, diuretics/weight-reducing drugs and thyroid hormones, and type of sports. The factors with a p-value below 0.2 were further evaluated in a multivariate logistic regression model. Risks were expressed as odds ratios (ORs) with 95% confidence intervals (95% CIs). P values less than 0.05 were considered significant. The SPSS program (Version 10) was used for all analysis.

## Results

A total of 788 individuals participated in the study. The age ranged from 13 to 37 (mean = 21.1, SD = 4.5). The mean age in the Amenorrhea/Oligomenorrhea group 20.1 ± 4.4, and in the eumenorrhea group 21.1 ± 4.5 was slightly different but was not statistically significant (T test: t = 0.417, p = 0.677). Table 1 presents the baseline characteristics of the study population. The mean BMI were 21.2 ± 2.9 (ranged 13.2 to 41.0). The mean BMI was not different between two groups; 21.9 ± 4.0, and 21.1 ± 2.7 in the Amenorrhea/Oligomenorrhea and eumenorrhea group respectively.

**Table 1:** Baseline characteristics of study population

	**Amenorrhea/Oligomenorrhea group (n = 71)**	**Eumenorrhea group (n = 717)**
BMI < 20	23 (32.9)	245 (34.9)
BMI 20–25	36 (51.4)	398 (56.7)
BMI > 25	11 (15.7)	59 (8.4)
		
Sport intensity, mean (SD) (hours/week)	14.35 (11.44)	12.64 (10.59)
		
**Level of education**		
High School undergraduate	16 (22.5)	161 (22.8)
High School graduate	26 (36.6)	242 (34.2)
Bachelors degree	29 (40.8)	304 (43.0)
**Types of sport**		
Other sports (reference)	36 (50.7)	438 (61.1)
subject scored	-	29 (4.0)
endurance sports	9 (12.7)	38 (5.3)
body contour	2(2.8)	31 (4.3)
weight categories	24 (33.8)	181 (25.2)
**History of Drug intake**		
OCPs	51 (71.8)	654 (91.2)
Diuretics & weight-reducing drugs	1 (1.4)	16 (2.2)
Thyroid hormones	-	15 (2.1)

Seventy one (9.0%) individuals had amenorrhea or oligomenorrhea, which oligomenorrhea occurred in 4.2%of participants. Secondary amenorrhea reported in 4.8%, and no cases of primary amenorrhea detected.

The mean age of menarche (768 cases) was 13.7 ± 1.6 years, ranged from age of 9 – 20. The mean age of menarche in the Amenorrhea/Oligomenorrhea group was 13.8 ± 1.9 and in the eumenorrhea group 13.7 ± 1.6, which was not statistical significant difference (T test: t = 0.143, p = 0.887).

Late-onset menarche occurred in 3.4% (26) of participants, of which 7.2% was in the Amenorrhea/Oligomenorrhea group and 3% in eumenorrhea group. This difference was statistically significant (Table 2).

**Table 2:** association between Amenorrhea/Oligomenorrhea and risk factors

	**OR (95% CI)** **Univariate Regression analysis**	**OR (95% CI)** **Multivariate Regression analysis**
Age < 20 years	1.58(1–2.57)	2.67 (1.47–4.85)*
BMI 20–25 (reference)		
BMI < 20	1.04(0.60–1.79)	0.88 (0.49 – 1.60)
BMI > 25	2.06(1.0–4.27)	1.48(0.62–3.53)
Sport intensity (hours per week)	1.01 (0.99–1.03)	1.02 (0.99–1.04)
Late-onset Menarche	2.52(0.92–6.91)	3.32(1.04–10.51)*
Use of OCPs	4.07(2.28–7.25)	6.17(3.00–12.69)*
Types of sport		
Other sports (reference)	1	1
subject scored	NA	NA
endurance sports	2.89(1.29–6.43)	2.89(1.22–6.84)*
body contour	0.79 (0.18–3.41)	0.20 (0.02–1.71)
weight categories	1.61(0.94–2.78)	2.09(1.15–3.82)*

All analyses were evaluated using the Logistics regression model.

All variable with a p-value < 0.2 in univarriate analysis entered in the multivariate model

* p-value < 0.05 ** p-value < 0.2

NA: Any participants in subject scored group reported Amenorrhea/Oligomenorrhea

Of the 71 participants with Amenorrhea/Oligomenorrhea whom has been evaluated for polycystic ovary disease, eleven participants diagnosed with this disease (15.5%).

### Association between Amenorrhea/Oligomenorrhea and potential risk factors

Table 2 summarizes the relationship of Amenorrhea/Oligomenorrhea to the age, BMI, Late-onset menarche, use of OCPs and type of sport and intensity of sport. Participants older than twenty years old were at less risk of Amenorrhea/Oligomenorrhea compared to participants younger OR; 2.67, 95%CI (1.47 – 4.85). Further evaluation in the multivariate model revealed the same results.

Being thin or fat showed no extra risk of Amenorrhea/Oligomenorrhea in this study (table 2). Late-onset menarche was a significant risk for Amenorrhea/Oligomenorrhea (table 2). Further adjustment for type of sport, age, BMI and use of OCPs did not change the results OR; 3.32, 95%CI (1.04 – 10.51).

In the Amenorrhea/Oligomenorrhea group, twenty one (28.2%) participants with Amenorrhea/Oligomenorrhea had used OCPs and one person (1.4%) has used diuretics and/or weight-reducing drugs. None of this group used thyroid hormones. In the eumenorrhea group, 63 (8.8%) participants used OCPs. Sixteen (2.2%), and 15 (2.1%) participants of this group used diuretics and/or weight-reducing drugs and thyroid hormones respectively. We detected no association between diuretics and/or weight-reducing drugs and amenorrhea/oligomenorrhea OR; 0.63, 95%CI (0.0.08 – 4.79), p-value = 0.652. We found a positive association between use of OCPs in the last year and Amenorrhea/Oligomenorrhea, (OR; 6.17 95%CI (3.00 – 12.69). Adjusted for other factors in the model, the risk of Amenorrhea/Oligomenorrhea was high in two types of sports; endurance sports (OR; 2.89, 95%CI (1.22 – 6.84) and weight categories (OR; 2.09, 95%CI (1.15 – 3.82).

## Discussion

These findings support the previous findings in the literature that the prevalence of amenorrhea and oligomenorrhea is high in athletes. Females practicing endurance and weight-class sports had a significantly higher risk of these disorders. There was also a positive association between the disorders and the following: age under 20 years old, late onset of menarche, and use of OCPs. Intensity of training sport or BMI were not risk factors. About one sixth of the athletes with amenorrhea/oligomenorrhea were diagnosed as PCOS.

To our knowledge, this is the first study in Iran to evaluate risk factors of amenorrhea and oligomenorrhea in athletes. A particular strength is its high response rate, which led to a large sample size. We invited all possible top female Iranian athletes from 34 sports federations, including female medalists in Tehran and female members of national teams. Very high percentages (95%) were included in this study. Another strength of this study is that it presents the prevalence of PCOS in women athletes, which has not been evaluated earlier.

Like earlier studies, we demonstrate that prevalence of amenorrhea/oligomenorrhea in athletes is high. Having compared the prevalence of amenorrhea/oligomenorrhea in our study with the prevalence in the general population reported in a review by Pfeifer and Patrizio [4], we conclude that these disorders are more frequent in athletes. However, as there are no reliable data on prevalence of amenorrhea/oligomenorrhea in the Iranian general population, we have not been able to compare our data with those of our population.

Like other studies [1,9,10], we show that amenorrhea/oligomenorrhea occurred significantly more in weight-class and endurance sports. The probable reason lies in weight-class athletes' focus on leanness and low body weight. They believe that the chance of success is higher if they can participate in the lowest possible weight category. This very belief leads to increased efforts to losing weight. To increase their performance, athletes in endurance sports tend to lose weight.

Unlike other study [9] and a report by the IOC [1], we detected no association between aesthetic sports and amenorrhea/oligomenorrhea. The disparity is probably explained by the fact that only two aesthetic sports were represented in our study: aerobic sport and vosho. None of the participants in this group reported any amenorrhea or oligomenorrhea. No participants did ballet, figure skating, or synchronized swimming. Menarche had not started in any of the gymnasts who responded, who were therefore not included in this study.

In recent years, some studies have reported significant relationships between menstrual disorders and late-onset menarche in athletes. In 2007, Micklesfield et al. studied menstrual disorders in 613 marathon and half-marathon runners in South Africa [11]. As in our study, they showed that onset of menarche was later in the group with amenorrhea/oligomenorrhea than in the group with regular menstruation. Menstrual disorders and late onset menarche both seem to have caused by intensive training and inadequate energy intake [12]

Our study also shows that participants aged under 20 reported more amenorrhea/oligomenorrhea than older participants. Readers are reminded that we excluded all participants who were in their first year of menarche and who had reported amenorrhea/oligomenorrhea, which we considered to be a physiological event. The experience of Iranian obstetricians is that these disorders are more likely to occur before the age of 20, even in girls who practice no sport. Their higher prevalence in athletes younger than 20 years seems to be due not to sporting activities, but to the immaturity of the girls' reproductive systems.

Although it has been shown that a certain percentage of fat mass is required for menstruation to begin and to continue regularly, no specific percentages have been reported [13,14]. This number seems to vary from person to person. Unlike other studies, we found no significant relationship between amenorrhea/oligomenorrhea and BMI (BMI < 20 and BMI > 25) [15]. The explanation may lie in the fact that many aesthetic sports which require extreme leanness (such as ballet, dance, figure-skating, and synchronized swimming) are not practiced in Iran. Moreover, the criticism of the use of BMI for body composition on assessment are that it is a relatively poor predictor of body fat percentage and it results in inaccurate classifications (normal overweight, obese) for some individuals [15]. Unfortunately, due to feasibility reason, we were unable to use more advanced methods such as measurement of skin fold, or bioelectrical impedance analysis to estimate body fat percentage.

Studies by the International Olympic Committee have reported a higher prevalence of amenorrhea/oligomenorrhea in athletes who seek to improve their physical performance through strict dietary restrictions or through strenuous exercises intended to induce weight loss [1]. The study by Torstveit et al. also showed that weight-class athletes use pathologic methods of weight reduction more than other athletes [9]. Another studies also confirm that menstrual dysfunction is more prevalent among athletes who focus on losing weight and becoming thinner [16]. Although Torstveit et al. reported a relationship between weight-reducing drugs and amenorrhea/oligomenorrhea [9], we observed no such relationship in our study. This may have been due to small number of participants who reported using such drugs. It appears that some participants may have denied using weight-reducing drugs out of fear of expulsion from the team, and out of fear of their parents' and coach's objections, etc.

Although, unlike Drinkwater et al. [17], we found no association between amenorrhea/oligomenorrhea and the intensity with which sports were practised, our results agreed with those of two other studies [18,19], which reported no difference between training intensity among amenorrhea and eumenorrhea athletes. Because we had no clear or reliable reports of abrupt onset of intense training, we could not evaluate whether there was any association between the onset of amenorrhea/oligomenorrhea and a sharp increase in sport intensity.

A review by Loucks et al.[20] in 2006 stated that the prevalence of PCOS in women athletes has never been assessed or reported. To our knowledge, our study is therefore the first on the prevalence of PCOS in women athletes. However, we should remind readers that we assessed PCOS in any participants with clinical symptoms of amenorrhea/oligomenorrhea, but not in those who had no mentioned of these disorders. Because some PCOS cases might show any clinical manifestation, we may have underestimated the prevalence of PCOS.

Haberland et al. reported in a survey in 1995 that over 90% of sports medicine doctors or family physicians prescribed estrogen replacement to treat athletic amenorrhea [21] The positive association we found between amenorrhea/oligomenorrhea and the use of OCPs might therefore be due to the prescription of OCPs for treating menstrual irregularities.

It should be pointed out that this study evaluated Iranian female athletes in organized sport federations. As stated above, the variety and frequency of these sports was not necessarily the same as those of leading women's sports in other countries: for example, ballet, figure skating, and synchronized swimming are not represented in any sport club in Iran. Although women's sports have been brought some difficulties by Islamic dress codes, they are nonetheless progressing slowly and steadily. As the number of professional women's clubs is very low, there are not many high-level competitions; similarly, international competition is subject to considerable limitation. We believe that the difference between our results and those of studies in non-Islamic countries are partly a consequence of these differences.

Future studies among Iranian female athletes should examine preventable risks, such as inappropriate dietary restrictions, pathologic methods of weight loss, psychological and physical stress, and athlete's personality types.

## Conclusion

In conclusion, to increase awareness risks of menstrual disorder-related exercise, it is necessary to design complete and comprehensive education programs for female athletes, their parents, their coaches, and the relevant authorities. There should be a greater focus on weight-class and endurance sports in Iran, and especially on their associations with the so-called female athlete triad (eating disorders, amenorrhea and osteoporosis). Since many athletes deny eating disorders, and since diagnosis of osteoporosis needs paraclinical investigation, amenorrhea/oligomenorrheaprovides the best starting point for detecting this triad. On the other hand, these disorders can themselves lead to other disorders, such as osteoporosis and stress fracture. Athletes would be greatly benefited by greater general awareness about the complications of amenorrhea/oligomenorrhea.

## Abbreviations

**ACSM**: American College of Sports Medicine; **PCOS**: Poly Cystic Ovarian Syndrome; **OCP**: Oral contraceptive; **FSH**: Serum follicle stimulating hormone; **LH**: Luteinizing hormone; **TSH**: Thyroid Stimulating Hormone; **DHEA**: Dehydroepiandrosteron.

## Competing interests

The authors declare that they have no competing interests.

## Authors' contributions

HD conceived of the study, and participated in its design, coordination and drafted the manuscript. MR participated in its design and coordinated the study. AA participated in its design and carried out medical examination. TA participated in its design, coordination and drafted the manuscript. SD performed the statistical analysis and drafted the manuscript. All authors read and approved the final manuscript.
